# Psychosocial Risk Factors Identified During the Child and Adolescent Crisis Care Program Intervention

**DOI:** 10.1192/j.eurpsy.2026.11162

**Published:** 2026-07-31

**Authors:** C. Diaz Tellez, I. Quiñoa Jimenez, M. Artigas Dorado, R. M. Calvo Escalona

**Affiliations:** Hospital Clinic, Barcelona, Spain

## Abstract

**Introduction:**

Globally, crisis services aim to prevent emergency visits for children and adolescents, provide appropriate evaluation and treatment, and ensure coordination with outpatient care. These programs often involve intensive interventions in community and home settings. This poster presents the implementation of the *Child and Adolescent Crisis Care Program*at Hospital Clínic de Barcelona, designed to deliver home-based and community care. Eligible patients include those with suspected or confirmed mental disorders or acute psychopathological crises, psychosocial risk, or social complexity limiting access to standard care.

**Objectives:**

To analyze the impact of the home-based intensive care program on family functioning and to identify family profiles, commitment, and adherence during the therapeutic process.

**Methods:**

A retrospective study of patients treated and discharged during the program’s first two years (November 2022–November 2024).

**Results:**

Fifty-one cases were attended. The most frequent psychosocial risk factors were inadequate supervision (53%), parent-child conflict (39%), physical abuse (33%), and psychological abuse (31%). Post-intervention, significant improvement was observed in perceived family functioning (p<0.001), including perceived support, intrafamily communication, joint decision-making, time spent together, and perceived esteem (all p<0.001).

Challenges during intervention included partial adherence to scheduled visits (98%) and limited willingness to engage in treatment (87.8%). The high complexity detected often required activation of child protection services.

**Conclusions:**

This program provides an effective response for cases with high clinical and sociofamilial complexity. Its multidisciplinary, intensive, and home-based approach offers a comprehensive and realistic view of acute situations and family dynamics that other settings cannot achieve. Findings have led to the development of a new therapeutic line: parent-focused group interventions to address identified difficulties.

**Disclosure of Interest:**

None Declared

